# Development of an Aerobic Glycolysis Index for Predicting the Sorafenib Sensitivity and Prognosis of Hepatocellular Carcinoma

**DOI:** 10.3389/fonc.2021.637971

**Published:** 2021-05-18

**Authors:** Yu Pan, Geng-yuan Hu, Shi Jiang, Shun-jie Xia, Hendi Maher, Zhong-jie Lin, Qi-jiang Mao, Jie Zhao, Liu-xin Cai, Ying-hua Xu, Jun-jie Xu, Xiu-jun Cai

**Affiliations:** ^1^Department of General Surgery, Sir Run-Run Shaw Hospital, Zhejiang University, Hangzhou, China; ^2^Key Laboratory of Laparoscopic Technology of Zhejiang Province, Hangzhou, China; ^3^Zhejiang Minimal Invasive Diagnosis and Treatment Technology Research Center of Severe Hepatobiliary Disease, Hangzhou, China; ^4^Zhejiang Research and Development Engineering Laboratory of Minimally Invasive Technology and Equipment, Hangzhou, China; ^5^Zhejiang University Cancer Center, Hangzhou, China; ^6^School of Medicine, Zhejiang University, Hangzhou, China; ^7^Department of Gastrointestinal Surgery, Shaoxing People's Hospital, Shaoxing Hospital of Zhejiang University, Shaoxing, China; ^8^Department of Oncology, Sir Run-Run Shaw Hospital, Zhejiang University, Hangzhou, China

**Keywords:** hepatocellar carcinoma, aerobic glycolysis, Sorafenib, biomarker, prognosis

## Abstract

Hepatocellular carcinoma (HCC) is a deadly tumor with high heterogeneity. Aerobic glycolysis is a common indicator of tumor growth and plays a key role in tumorigenesis. Heterogeneity in distinct metabolic pathways can be used to stratify HCC into clinically relevant subgroups, but these have not yet been well-established. In this study, we constructed a model called aerobic glycolysis index (AGI) as a marker of aerobic glycolysis using genomic data of hepatocellular carcinoma from The Cancer Genome Atlas (TCGA) project. Our results showed that this parameter inferred enhanced aerobic glycolysis activity in tumor tissues. Furthermore, high AGI is associated with poor tumor differentiation and advanced stages and could predict poor prognosis including reduced overall survival and disease-free survival. More importantly, the AGI could accurately predict tumor sensitivity to Sorafenib therapy. Therefore, the AGI may be a promising biomarker that can accurately stratify patients and improve their treatment efficacy.

## Introduction

Globally, hepatocellular carcinoma (HCC) is the sixth most commonly diagnosed cancer and the fourth leading cause of cancer-related deaths (1). Despite advances in the treatment of HCC, its prognosis remains unsatisfactory, with a 5-year overall survival (OS) rate of 25–55% (2–4). Local recurrence, distal metastasis, and resistance to conventional therapy are the leading causes of HCC progression into late-stage cancer with limited treatment options. Genetic mutations, chromosomal instability, epigenetic changes, and molecular signaling pathway dysregulation are reported causes of hepatocellular carcinogenesis (5). Therefore, advances in the field of molecular oncology are urgently required to improve the prognosis of HCC.

Sorafenib is an oral multitargeted drug that inhibits the activity of several tyrosine kinases (6). Thus, Sorafenib can effectively suppress angiogenesis and cancer proliferation and induce tumor cell apoptosis. Since it was first approved by the US Food and Drug Administration in 2007 as the first-line treatment for advanced HCC, Sorafenib has shown favored clinical benefits. In the Sorafenib Hepatocellular Carcinoma Assessment Randomized Protocol (SHARP) trial, patients who received Sorafenib therapy showed significantly higher median OS compared with the control group (10.7 vs. 7.9 months, respectively) and a 31% reduction in the risk of death (7). In the Asia-Pacific trial, Sorafenib provided a clinical benefit, extending the median survival benefit by 2 months (8). Currently, Sorafenib is still applied as the first-line therapy for advanced HCC patients; even several other tyrosine kinases inhibitors have been evaluated by comparing them to Sorafenib, which did not demonstrate an improvement of prognosis (9–11). However, a large number of patients with HCC show poor response to Sorafenib due to the heterogeneity of the disease and the complex tumor-associated molecular signaling, which lacks generally accepted predictive biomarkers. Furthermore, primary and acquired resistance to Sorafenib are commonly reported and limit the clinical advantages of the drug (12–15).

The Warburg phenotype is a common hallmark of cancer cells, characterized by enhanced glycolysis, even under physiological oxygen conditions (16, 17). By shifting glucose metabolism from oxidative respiration to aerobic glycolysis, tumor cells display enhanced glucose metabolism for producing efficient energy and various metabolic intermediates, which are indispensable for the synthesis of macromolecules and new organelles. Several oncogenes, including Ras, Myc, and HIF1, were reported to drive metabolic adaptations toward aerobic glycolysis (18, 19). Enhanced aerobic glycolysis has been demonstrated to exhibit consistent prognostic patterns and is associated with Sorafenib resistance (20). Increased expression of aerobic glycolysis-related genes, including solute carrier family 2 member 1 (SLC2A1), solute carrier family 2 member 2 (SLC2A2), glucose-6-phosphate dehydrogenase (G6PD), glypican 1 (GPC1), procollagen-lysine, 2-oxoglutarate 5-dioxygenase 2 (PLOD2), and lactate dehydrogenase A (LDHA), has been reported to be associated with aggressive HCC (21–29). Accelerated glucose uptake and lactate synthesis were observed as responses to Sorafenib treatment (30–32). New therapeutic approaches have been reported to attenuate Sorafenib resistance by inhibiting key glycolytic enzymes including 6-phosphofructo-2-kinase/fructose-2,6-biphosphatase 3 (PFKFB3), hexokinase 2 (HK2), and pyruvate kinase M1/2 (PKM2) (33–36). Tumor metabolic heterogeneity is reported to be relevant to tumor subtypes and prognosis (37), but whether heterogeneity in distinct metabolic pathways can be used to stratify HCC into clinically relevant subgroups has not been well-established.

Aerobic glycolysis is a complex biological process involving numerous genes. Thus, constructing a gene signature based on multiple glycolysis-related genes is supposed to be more suitable to represent the aerobic glycolysis pathway than single gene. The development of genomic techniques has unveiled extensive biological information that can be used to explore the underlying mechanisms of tumorigenesis and progression. In this study, we constructed a model named aerobic glycolysis index (AGI) to evaluate the signal of aerobic glycolysis, by utilizing genomic data of hepatocellular carcinoma from The Cancer Genome Atlas (TCGA) project. The AGI was calculated based on the expression of 14 glycolysis-related genes (SLC2A1, SLC2A2, G6PD, LDHA, GPC1, HMMR, PLOD2, GOT2, STC2, CENPA, RARS1, HOMER1, SRD5A3, and TKTL1). The abbreviations list and their expansions for these glycolytic genes are summarized in Supplementary Table 1. Then, we assessed whether the AGI was a predictive marker for the prognosis of HCC and sensitivity to Sorafenib. Finally, we used *in vitro* experiments to confirm that AGI was associated with Sorafenib resistance. Significantly, we established a methodology to quantify aerobic glycolysis signaling. The AGI was found to be a robust prognostic biomarker of HCC and a predictive factor of the response to Sorafenib.

## Materials and Methods

### Specimen Collection and RNA Sequencing

In total, 102 pairs of formalin-fixed paraffin-embedded HCC and corresponding normal tissue specimens from the Sir Run Shaw Hospital (SRRSH) were collected. This research was approved by the Institutional Review Board of the SRRSH of Zhejiang University. The patients provided informed consent for the use of their specimens. For RNA sequencing, TRIzol (Invitrogen, USA) was used to extract total RNA. After checking the RNA purity, integrity, and concentration, RNA sequencing was performed on an Illumina platform.

### Data Acquisition and Processing

Fragments per kilobase of transcript per million mapped reads (FPKM) RNA-seq data and the clinical characteristics of TCGA samples were downloaded from the UCSC Cancer Browser database. Gene mutation data and copy number information of the TCGA samples were acquired from the cBioPortal database. RNA-seq and clinical data of the LIRI-JP cohort were downloaded from the HCCDB database. The GSE14520, GSE25097, GSE36376, GSE64041, GSE76427, GSE109211, and GSE73571 expression profile was obtained from the Gene Expression Omnibus (GEO) database. The proteomics data of TCGA samples were downloaded from the TCPA database. Drug sensitivity data of HCC cell lines were obtained from the Genomics of Drug Sensitivity in Cancer (GDSC) database and Cancer Cell Line Encyclopedia (CCLE) database. The patient characteristics of TCGA, LIRI-JP, GSE14520, and SRRSH datasets are summarized in Supplementary Table 2.

### Development of the AGI

Univariate Cox regression was applied to detect the aerobic glycolysis genes related to prognosis. The least absolute shrinkage and selection operator (LASSO) Cox regression model was performed to determine the coefficients for model construction with an optimal log λ (38). The AGI was established with the following formula: Risk score = expression of Gene 1 × β1 + expression of Gene 2 × β2+…expression of Gene n × βn (β was the weighted coefficient of each gene). For gene expression measured by quantitative real-time PCR (qPCR), the AGI was calculated as the method described by Zheng et al. (39). Total RNAs from cells were extracted using TRIzol (Invitrogen, USA) according to the manufacturer's instructions. Complementary DNA (cDNA) was synthesized using Hifair® II 1st Strand cDNA Synthesis SuperMix for qPCR (Yeasen, Shanghai, China). qPCR was performed using Hieff UNICON® qPCR SYBR Green Master Mix (Yeasen, Shanghai, China). Measurement was carried out by Roche LightCycler 480. Analysis was carried out using the ^ΔΔ^Ct method. All assays were performed in triplicates, and results were plotted as the mean ± SD. Primer sequences are listed in Supplementary Table 3.

### Gene Set Enrichment Analysis and Gene Set Variation Analysis

Datasets were divided into two groups according to tissue types or AGI scores. Annotated gene sets were downloaded from the MSigDB database. Gene Set Enrichment Analysis (GSEA) was performed using the R package “GSEAbase.” Annotated drug sets were downloaded from the DSigDB database. Gene Set Variation Analysis (GSVA) was performed using the R package “GSVA.”

### Cell Culture of Liver Cancer Cell Lines

Liver cancer cell lines (SK-Hep-1, Huh7, HepG2, HCCLM3) were purchased from the American Tissue Culture Collection (Manassas, VA, USA) and cultured in accordance with the recommended guidelines. Sorafenib-resistant HCC cell lines were cultured with Sorafenib as previously reported (40, 41).

### Cell Viability Test

Cells were seeded in 96-well plates in replicates of three. After incubation with Sorafenib for 48 h, cell viability analysis was performed using the Cell Counting Kit-8 (CCK-8) (Yeasen, Shanghai, China).

### Apoptosis Assay

Cells were seeded in six-well plates and mock treated or treated with drugs [Sorafenib, 2-deoxy-D-glucose (2-DG), or a combination of Sorafenib and 2-DG] for 48 h before apoptosis assays. Cell apoptosis was determined using the PI/annexin V-FITC Apoptosis Kit (MULTI SCIENCES, Hangzhou, China).

### Transwell Assay

Cells (1 × 10^5^) in serum-free medium were seeded into the upper chambers of Transwell (Corning, Corning, NY, USA), and medium with 10% fetal bovine serum (FBS) was seeded into the lower chambers for 24 h in a humidified incubator at 37°C in 5% CO_2_. The cells remaining in the upper chamber were carefully removed using a cotton swab, and cells that migrated to the lower membrane surface were fixed in 4% paraformaldehyde and stained with crystal violet. The experiments were repeated three times.

### Glucose Consumption and Lactic Acid Assays

Cells were seeded into six-well plates at a density of 1 × 10^6^ cells and cultured overnight. Glucose consumption and lactic acid production were detected using glucose assay kit (Solarbio® BC2500) and LA assay kit (Solarbio® BC2230) according to the instruction of the manufacturers, respectively. The experiments were repeated three times.

### Statistical Analysis

The univariate Cox regression, LASSO Cox regression model, and multivariate Cox regression model were performed. The OS and disease-free survival (DFS) were compared using the Kaplan–Meier method with the log-rank test. Non-parametric tests or Student's *t*-tests were used to determine the significance of the differences between the subgroups and clinicopathological characteristics. Spearman's correlation test was used to assess the relationship between the AGI and biological pathways and clinicopathological parameters. Statistical analyses were performed using R software (Version 3.6.0). A P < 0.05 was considered statistically significant, and all *P*-values were two-tailed.

## Results

### Establishment of the AGI

**Figure 1:**
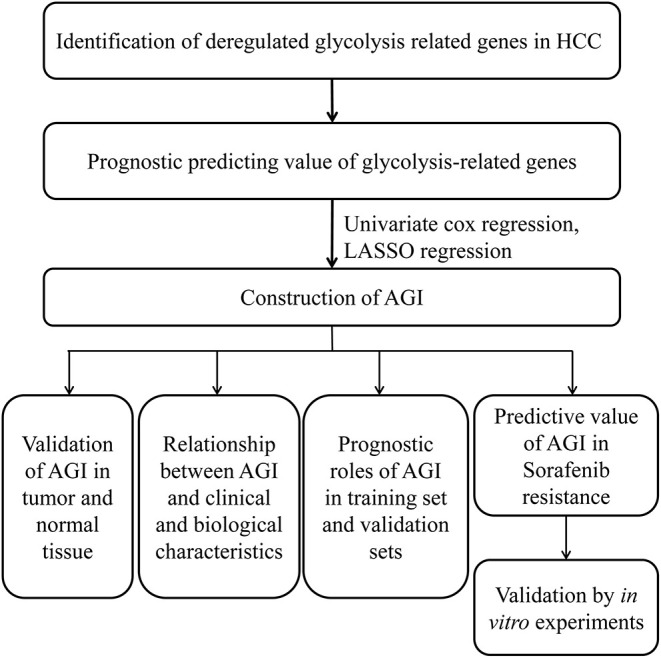
Flowchart presenting the establishment and validation of the gene signature.

**Figure 2:**
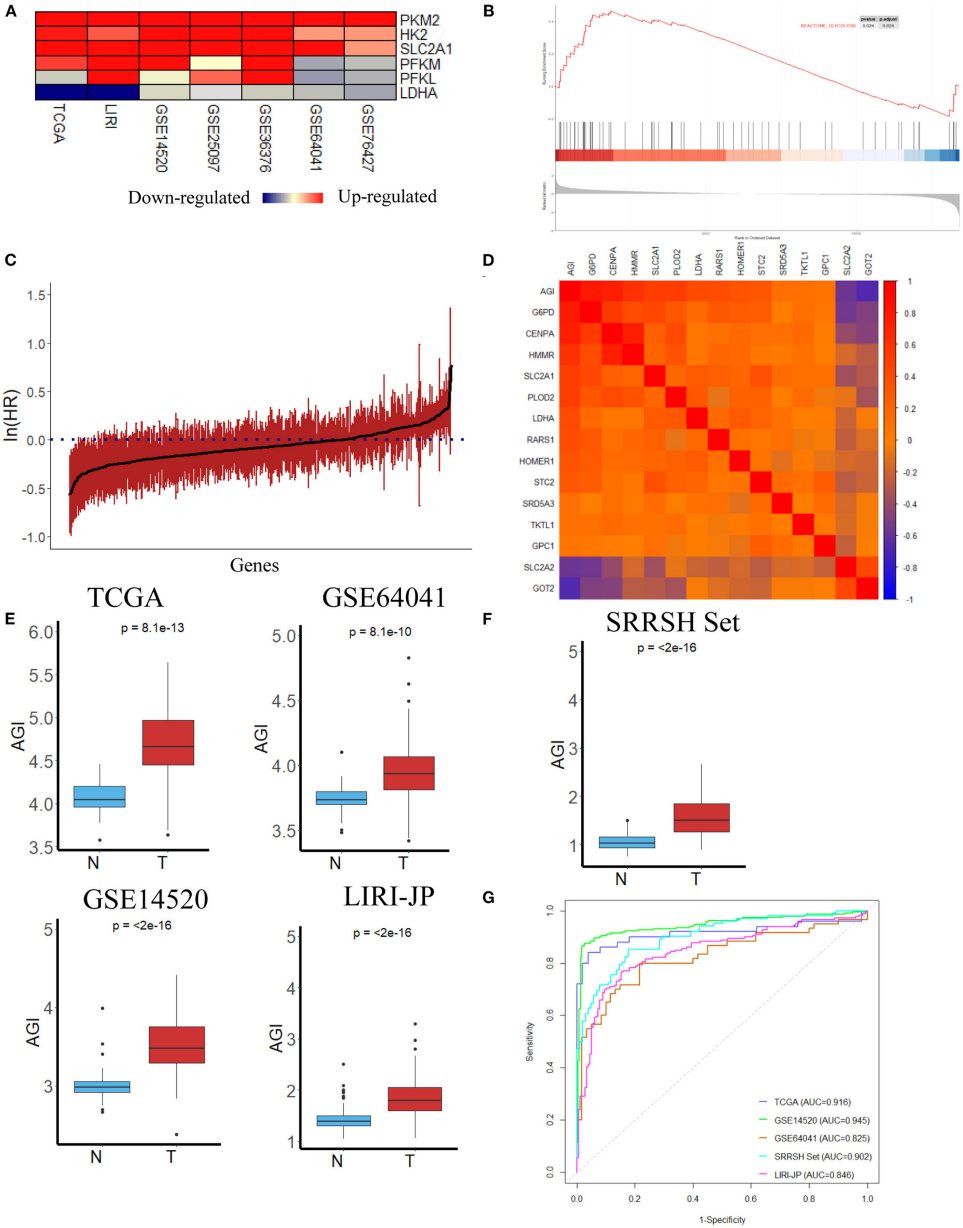
Construction of the aerobic glycolysis index (AGI) model and validation of the AGI in tumor and normal tissues. **(A)** Heatmap of glycolysis-related gene expression in different datasets. **(B)** Gene Set Enrichment Analysis (GSEA) of the glycolysis pathway in GSE14520. **(C)** Bar plot showing the hazard ratio of glycolysis-related genes in The Cancer Genome Atlas (TCGA) cohort using the univariate Cox regression. The bars represent the 95% CI. **(D)** Correlation between the AGI and the selected signature genes in the TCGA cohort. **(E)** Boxplots showing AGI differences in normal and tumor tissues in the TCGA, GSE64041, GSE14520 and LIRI JP datasets. **(F)** Boxplots showing AGI differences in normal and tumor tissues in the SRRSH set. **(G)** Receiver operating characteristic (ROC) curves for tissue type prediction using the AGI as the predictor.

This study was conducted according to the flow chart shown in Figure 1. Initially, several key genes of glycolysis were observed to be commonly upregulated in tumor tissues compared with normal tissues in a series of datasets, including TCGA, LIRI-JP, GSE14520, GSE25097, GSE36376, GSE64041, and GSE76427 (Figure 2A). The GSEA results revealed that the glycolysis signaling pathway was significantly enriched in HCC tumor tissues compared with normal tissues (Figure 2B). These results suggested that the glycolysis signaling pathway may contribute to malignant tumor phenotypes. Because aerobic glycolysis is a complex biological process involving hundreds of genes, using a gene signature comprising multiple genes can predict the tumor characteristics and prognosis more accurately than a single gene. The univariate Cox regression analysis was conducted to determine the prognostically relevant genes related to the aerobic glycolysis, and 80 relevant genes were identified (Figure 2C). To further simplify the gene signature of aerobic glycolysis, LASSO regression was performed based on these prognostically relevant genes. Finally, 14 genes were selected to establish the AGI according to the partial likelihood deviance method with an optimal log λ (Supplementary Figures 1, 2). Additionally, as shown in Figure 2D, the correlations between the AGI and selected aerobic glycolysis-related genes were statistically significant. Furthermore, the correlations between the AGI and several important genes coding the rate-limiting enzymes of glucose metabolism were examined. The results demonstrated that the AGI correlated closely to these genes (HK2, *r* = 0.43, *P* < 2.2e^−16^; PFKP, *r* = 0.39, *P* = 1.4e^−14^; PFKFB3, *r* = 0.27, *P* = 2e^−7^; PKM2, *r* = 0.66, *P* < 2e^−16^), as shown in Supplementary Figure 3.

After the establishment of the AGI, we first tested the capacity of AGI as an acceptable indicator of aerobic glycolysis. The AGI could distinguish HCC tumor tissues from normal tissue samples in the TCGA dataset that significantly higher AGI was observed in tumor samples, indicating enhanced aerobic glycolysis activity in these tumor samples (*P* = 8.1e^−13^, Figure 2E). To further validate the power of the AGI, we examined this index in three other HCC datasets. As shown in Figure 2E, a significantly higher AGI was observed in HCC tumor samples compared with normal tissue samples in all of three datasets (GSE64041, *P* = 8.1e^−10^; GSE14520, *P* < 2e^−16^; LIRI-JP, *P* < 2e^−16^). Furthermore, RNA sequencing data from our center (SRRSH) were applied and confirmed the reliability of AGI in distinguishing HCC tumor tissues from normal tissue samples (*P* < 2e^−16^) (Figure 2F). Then, the receiver operating characteristic (ROC) curve and area under the curve (AUC) scores were further evaluated to quantify the accuracy of the AGI to classify tumor and normal tissues. High prediction accuracies were achieved in all datasets, ruling out the possibility of over fitting (TCGA, AUC = 0.916; GSE64041, AUC = 0.825; GSE14520, AUC = 0.945; LIRI-JP, AUC = 0.846, SRRSH set, AUC = 0.902, Figure 2G).

### Association of the AGI With Genomic and Proteomic Alterations

**Figure 3:**
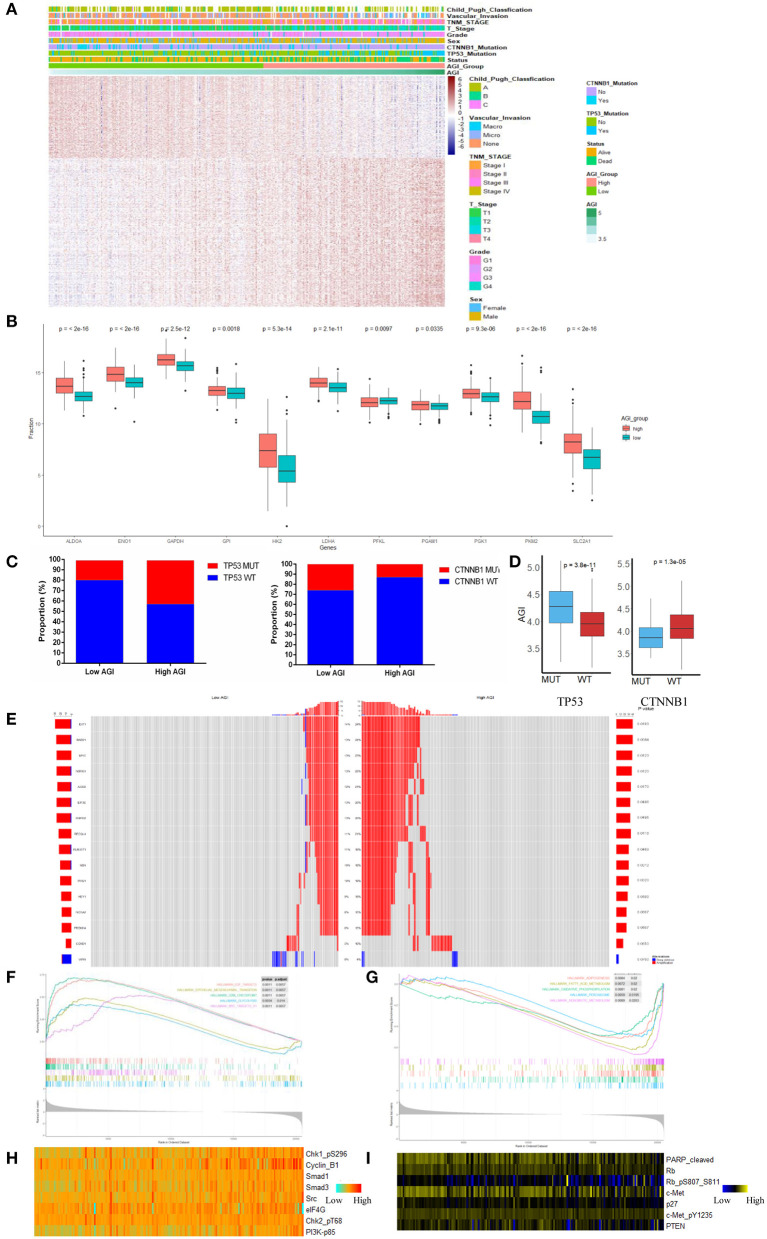
The landscape of biological processes and characteristics of the aerobic glycolysis index (AGI) subgroups. **(A)** Heatmap of common differentially expressed genes based on the expression data in the high and low AGI groups. **(B)** Box plots showing the expression of the selected glycolysis-related genes in The Cancer Genome Atlas (TCGA) cohort. **(C)** Proportion of TP53 and CTNNB1 mutations in the high and low AGI groups. **(D)** Box plots showing the AGI in patients with TP53 mutations and wild-type TP53 (left) and CTNNB1 mutations and wild-type CTNNB1 (right). **(E)** The oncoPrint of copy number variations was constructed in the high and low AGI subgroups. **(F)** Activated gene sets enriched in the high AGI subgroup. **(G)** Suppressed gene sets enriched in the high AGI group. **(H,I)** Proteins positively **(H)** and negatively **(I)** correlated with the AGI (P < 0.05 for all proteins) based on reverse-phase protein arrays analysis of 181 samples from the TCGA using Spearman's rank correlation.

Using the optimal cutoff value based on prognostic effects, the patients in the TCGA cohort were stratified into high and low AGI groups. The distribution of gene expression in the high and low AGI groups is represented in Figure 3A. In particular, we compared the genes encoding key proteins and enzymes of aerobic glycolysis, and most of them were highly expressed in the high AGI group, supporting the close correlation between aerobic glycolysis and the AGI (Figure 3B).

Previous reports demonstrated that aerobic glycolysis genes could be regulated by transcription factors such as p53, c-Myc, and HIF-1α. In our study, the high AGI group showed an increased proportion of TP53 mutations and a decreased proportion of CTNNB1 mutations (Figure 3C), which were the most common mutations in HCC. Accordingly, patients with a TP53 mutation showed a higher AGI than patients with wild-type TP53. Conversely, patients with a CTNNB1 mutation showed a lower AGI than patients with wild-type CTNNB1 (Figure 3D). Next, we investigated the distribution of copy number variations between the high and low AGI groups. The high AGI group showed an increased amplification frequency of Myc, AGO2, EXT1, RAD21, EIF3E, RSPO2, RECQL4, RUNX1T1, NBN, PAG1, and HEY1 (Figure 3E). These outcomes may provide novel ideas for investigating the mechanism of tumor aerobic glycolysis and copy number variation.

GSEA of the transcripts in the two groups revealed that gene sets considered to be markers of high malignancy were enriched in the high AGI group, including those E2F targets, Myc targets, epithelial–mesenchymal transition (EMT) regulators, and G2M checkpoints (Figure 3F and Supplementary Table 4). Conversely, gene sets related to oxidative phosphorylation, peroxisomes, xenobiotic metabolism, and other metabolic processes were enriched in the low AGI group (Figure 3G and Supplementary Table 4). As the EMT pathway enriched in the high AGI group, we were interested in the relationship between the AGI and tumor invasion capability. The AGI in four liver cancer cell lines (SK-hep-1, Huh7, HepG2, and HCCLM3) was calculated. The result demonstrated that cell lines with high AGI (SK-hep-1, Huh7, and HCCLM3) had increased glucose intake and lactic acid level as compared with the cell line with low AGI (HepG2). More importantly, the Transwell assay revealed that cell lines with high AGI exhibited the greater invasion capability (Supplementary Figure 4). Previous studies also have reported a close relationship between aerobic glycolysis and angiogenesis (42–44). In the present study, we compared the expression of several genes involved in angiogenesis between the high and low AGI groups. The results demonstrated that the high AGI group had increased expression of vascular endothelial growth factor A (VEGFA) and VEGFB as compared with the low AGI group (Supplementary Figure 5).

Further analysis of proteomic data revealed that the AGI was strongly correlated with the expression of cell-cycle-related proteins, including pChk1, pChk2, and CyclinB1, and other tumor hallmark proteins, such as eIF4G, pPI3K, Src, Smad1, and Smad3 (Figure 3H). Furthermore, the AGI was negatively correlated with the expression of Rb, phosphatase and tensin homolog (PTEN), cleaved poly(ADP-ribose) polymerase (PARP) (Figure 3I). Collectively, the analysis of gene mutations, copy number variations, classical signaling pathway gene sets, and proteomic data suggests that the AGI is associated with cell proliferation, tumor progression, and the inhibition of apoptosis in HCC.

### Correlation Between the AGI and Clinical Characteristics

**Figure 4:**
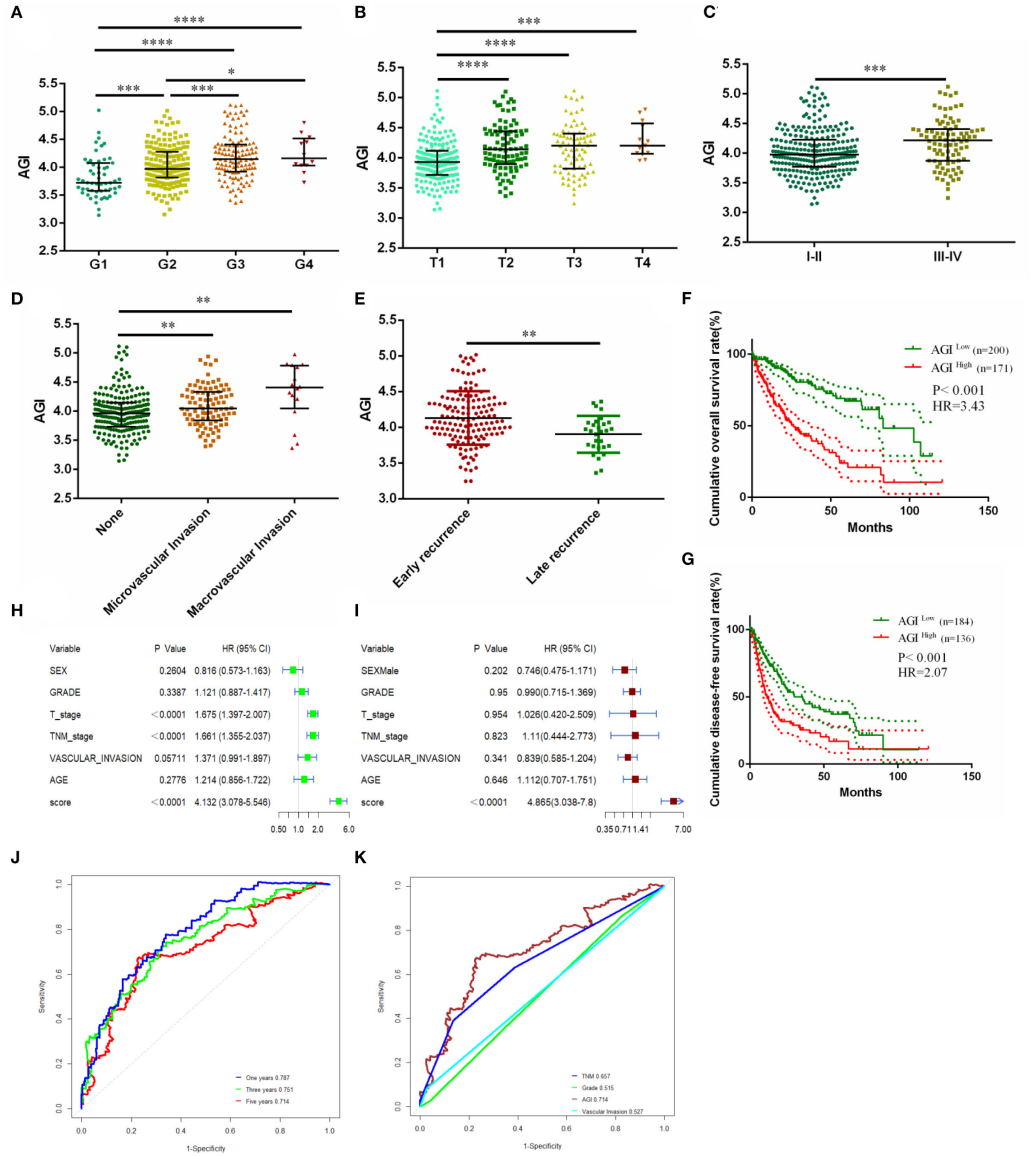
Clinicopathological significance and prognosis prediction value of the AGI. **(A)** Tumor differentiation grade. **(B)** T stage. **(C)** Tumor–node–metastasis (TNM) stage. **(D)** Vascular invasion status. **(E)** Recurrence status. **(F)** Kaplan–Meier plot analysis of overall survival (OS) in the high and low AGI groups. **(G)** Kaplan–Meier plot analysis of disease-free survival (DFS) in the high and low AGI groups. **(H,I)** Forest plot showing the prognostic value of the AGI and clinical characteristics using univariate **(H)** and multivariate **(I)** analysis. **(J)** Time-dependent receiver operating characteristic (ROC) analysis comparing the AGI in predicting the 1-, 3-, and 5-year OS. **(K)** Time-dependent ROC analysis comparing the AGI and clinical characteristics in 5-year OS. ^*^P < 0.05, ^**^P < 0.01, ^***^P < 0.001, ^****^P < 0.0001.

Our data showed that the AGI was closely associated with TP53, Myc, cell cycle, and EMT pathways, and therefore, we next examined whether the AGI is associated with tumor progression and metastasis. We first analyzed the relationship between the AGI and clinical characteristics of HCC patients. A significantly increased AGI was observed in patients with higher tumor grades (Figure 4A), advanced T stages, and tumor–node–metastasis (TNM) stages (Figures 4B,C) and vascular invasion (Figure 4D). Here, we defined early tumor recurrence as a tumor recurring within 2 years after primary treatment and late recurrence as cancer recurring after 2 years. The AGI was significantly higher in patients with early recurrence than in those with late recurrence (Figure 4E).

Patients with a higher AGI were associated with a worse prognosis in terms of OS [hazard ratio (HR), 3.43; *P* < 0.001; Figure 4F] and DFS (HR, 2.07; *P* < 0.001; Figure 4G). The univariate Cox regression analysis revealed that the AGI (HR, 4.132), T stage (HR, 1.675), and TNM stage (HR, 1.661) were risk factors of a worse HCC prognosis (Figure 4H), and the multivariate Cox regression analysis showed that the AGI was an independent risk factor of poor prognosis (HR, 4.865; Figure 4I). The ROC curve showed that the AGI could accurately predict the 1-year (AUC, 0.787), 3-year (AUC, 0.751), and 5-year OS (AUC, 0.714) of HCC (Figure 4J), which was superior to conventional clinical parameters such as the tumor grade, status of vascular invasion, and TNM stage (Figure 4K).

### Prognostic Value Validation of the AGI

**Figure 5:**
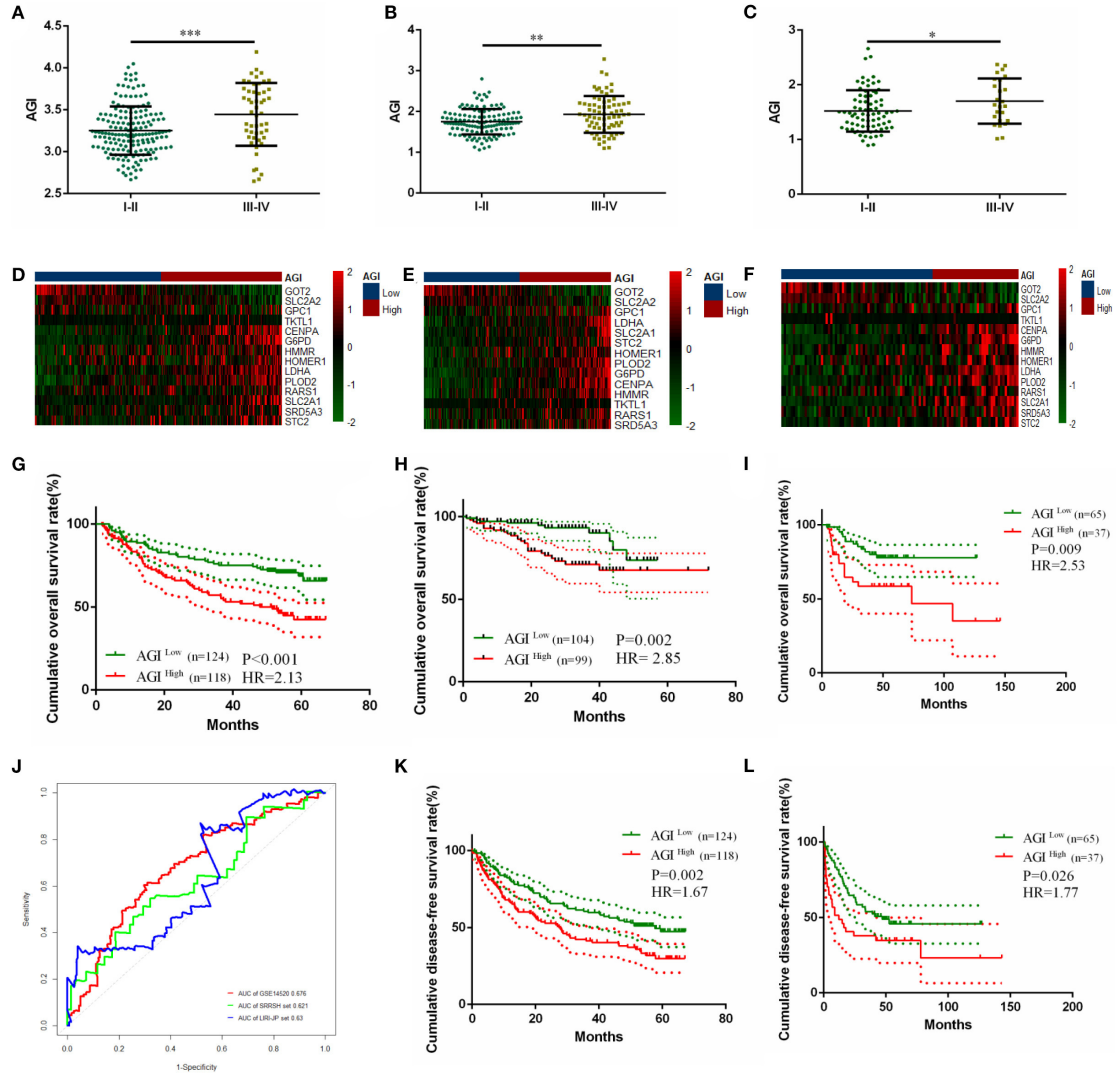
Validating the prognostically predictive value of the aerobic glycolysis index (AGI) in validation datasets. **(A–C)** Value of the AGI in different tumor stages in GSE14520 **(A)**, LIRI-JP **(B)**, and SRRSH set **(C)**. **(D–F)** The heatmap and distribution of the 14 AGI-related gene expression profiles in GSE14520 **(D)**, LIRI-JP **(E)**, and SRRSH set **(F)**. **(G–I)** Kaplan–Meier plot analysis of overall survival (OS) in the high and low AGI subgroups in GSE14520 **(G)**, LIRI-JP **(H)**, and SRRSH set **(I)**. **(J)** Time-dependent ROC analysis comparing the predictive value of the AGI for 5-year OS in the three datasets. **(K,L)** Kaplan–Meier plot analysis of disease-free survival (DFS) in the high and low AGI subgroups in GSE14520 **(K)** and SRRSH set **(L)**. *P < 0.05, ^**^P < 0.01, ^***^P < 0.001.

A significantly increased AGI was observed in patients with higher TNM stages in three independent datasets (Figures 5A–C). The prognostic value of the AGI in HCC was validated in three independent datasets, including GSE14520 from GEO, LIRC from ICGC, and the SRRSH set from our center. By dividing the datasets into two groups according to the AGI, the distribution of the gene expression profiles and status of patients were consistent with the AGI (Figures 5D–F). Similar to the TCGA dataset, the high AGI group showed worse OS (GSE14520: HR, 2.13; Figure 5G; LIRC: HR, 2.85; Figure 5H; SRRSH set: HR, 2.53; Figure 5I) and DFS (GSE14520: HR, 1.67; Figure 5K; SRRSH set: HR, 1.77; Figure 5L) compared with the low AGI group. The ROC curve demonstrated that the AUCs of the AGI in predicting the 5-year OS of GSE14520, LIRC, and SRRSH sets were 0.676, 0.630, and 0.621, respectively (Figure 5J), indicating a robust prognostic value of the AGI.

### AGI Predicts Sensitivity to Sorafenib in Both HCC Cell Lines and Patients

**Figure 6:**
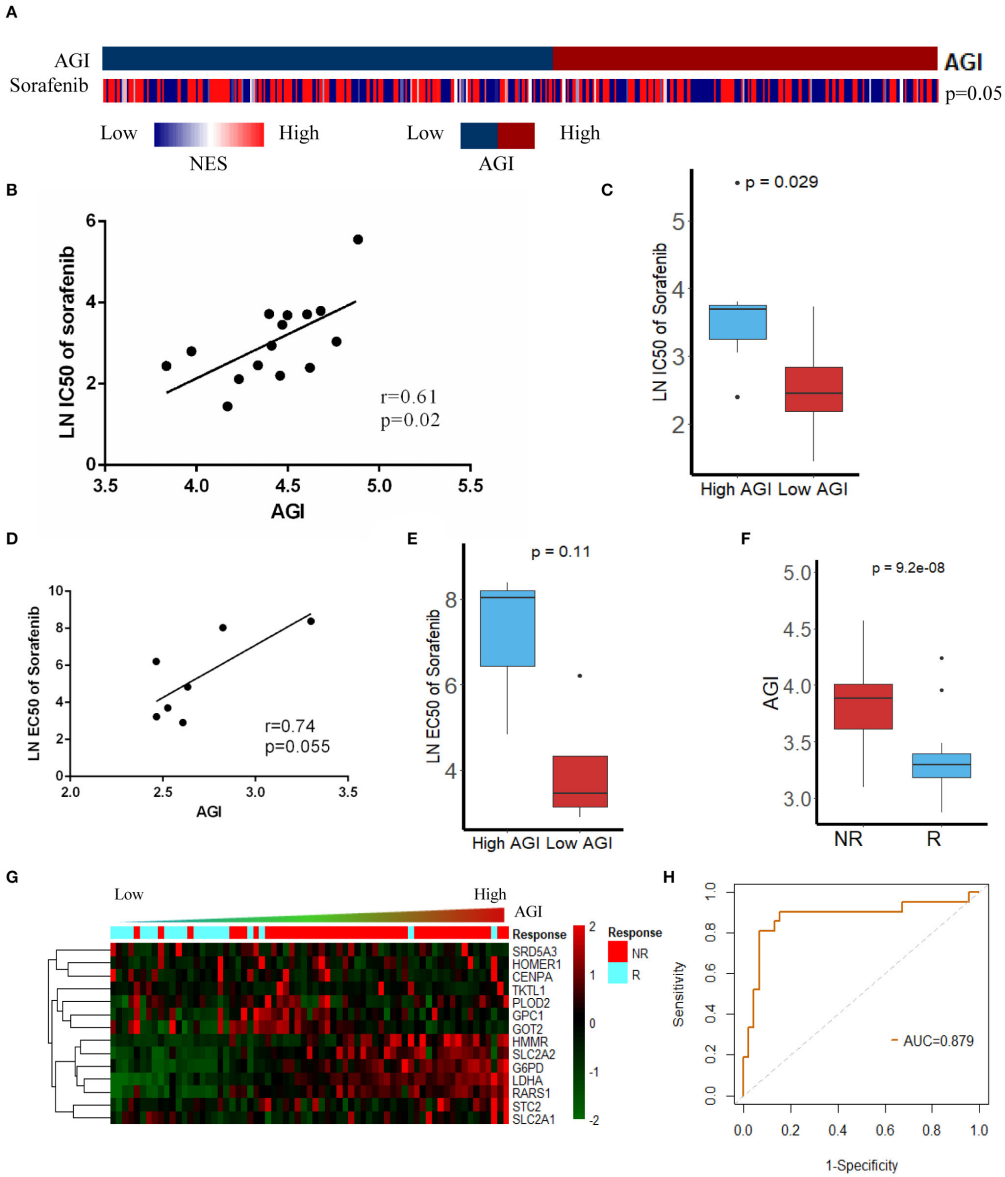
Association between the aerobic glycolysis index (AGI) and Sorafenib resistance. **(A)** The Gene Set Variation Analysis (GSVA) results showed that the Sorafenib sensitivity signature was enriched in patients with a low AGI. **(B)** AGI positively was correlated with the IC_50_ of Sorafenib in hepatocellular carcinoma (HCC) cell line data from the Genomics of Drug Sensitivity in Cancer (GDSC) database. **(C)** The IC_50_ of Sorafenib in HCC cell line data from the GDSC database with high and low AGIs. **(D)** AGI positively correlated with the EC_50_ of Sorafenib in HCC cell line data from the Cancer Cell Line Encyclopedia (CCLE) database. **(E)** EC_50_ of Sorafenib in HCC cell line data from the CCLE database with high and low AGIs. **(F)** Patient sensitive to Sorafenib presented significantly low AGI. **(G)** The heatmap and distribution of the 14 AGI-related gene expression profiles in GSE109211. **(H)** Receiver operating characteristic (ROC) analysis showed an area under the curve (AUC) of 0.879 for the AGI in predicting the response to Sorafenib.

Sorafenib is the first-line therapy for advanced HCC. The increased expression of aerobic glycolysis-related genes has been demonstrated to promote Sorafenib resistance (45). Our results showed the AGI was closely related to advanced tumor stages and poor tumor differentiation. Moreover, a high AGI was strongly correlated with EMT, Myc, and cell cycle pathways (Figure 3B), which had been reported to be associated with impaired Sorafenib sensitivity and a worse prognosis (45). We were interested in the relationship between the AGI and Sorafenib sensitivity and wondered if the AGI could be a potential biomarker to predict drug sensitivity. Thus, the GSVA was performed, and the results revealed that the low AGI group appeared to be more sensitive to Sorafenib (Figure 6A). Then, we verified the relationship between Sorafenib sensitivity and the AGI *in vitro* using the data from the GDSC database, which showed that HCC cell lines with a lower AGI were more sensitive to Sorafenib (Figure 6B). Furthermore, a positive correlation between the AGI and the natural logarithm of the IC_50_ was observed (*r* = 0.61, *P* = 0.02, Figure 6C). Similarly, using HCC cell line data from the CCLE database, cell lines with a higher AGI showed decreased Sorafenib sensitivity (Figure 6D), and a positive correlation between the AGI and the natural logarithm of the EC_50_ of Sorafenib was observed (Figure 6E). Although not statistically significant due to a limited sample size, the trends suggested a potential relationship between the AGI and Sorafenib sensitivity *in vitro*. We next calculated the AGI in patients who received Sorafenib therapy using the transcription data from GSE109211 (the STORM trial). The patients who did not respond to Sorafenib showed a higher AGI (Figure 6F) and upregulated expression of AGI-related genes (Figure 6G). The ROC curve of the AGI in predicting the sensitivity to Sorafenib retrieved an AUC of 0.879 (Figure 6H), indicating that the AGI is a reliable biomarker in selecting suitable patients for Sorafenib treatment.

### Increased AGI in Sorafenib-Resistant HCC Cell Lines

**Figure 7:**
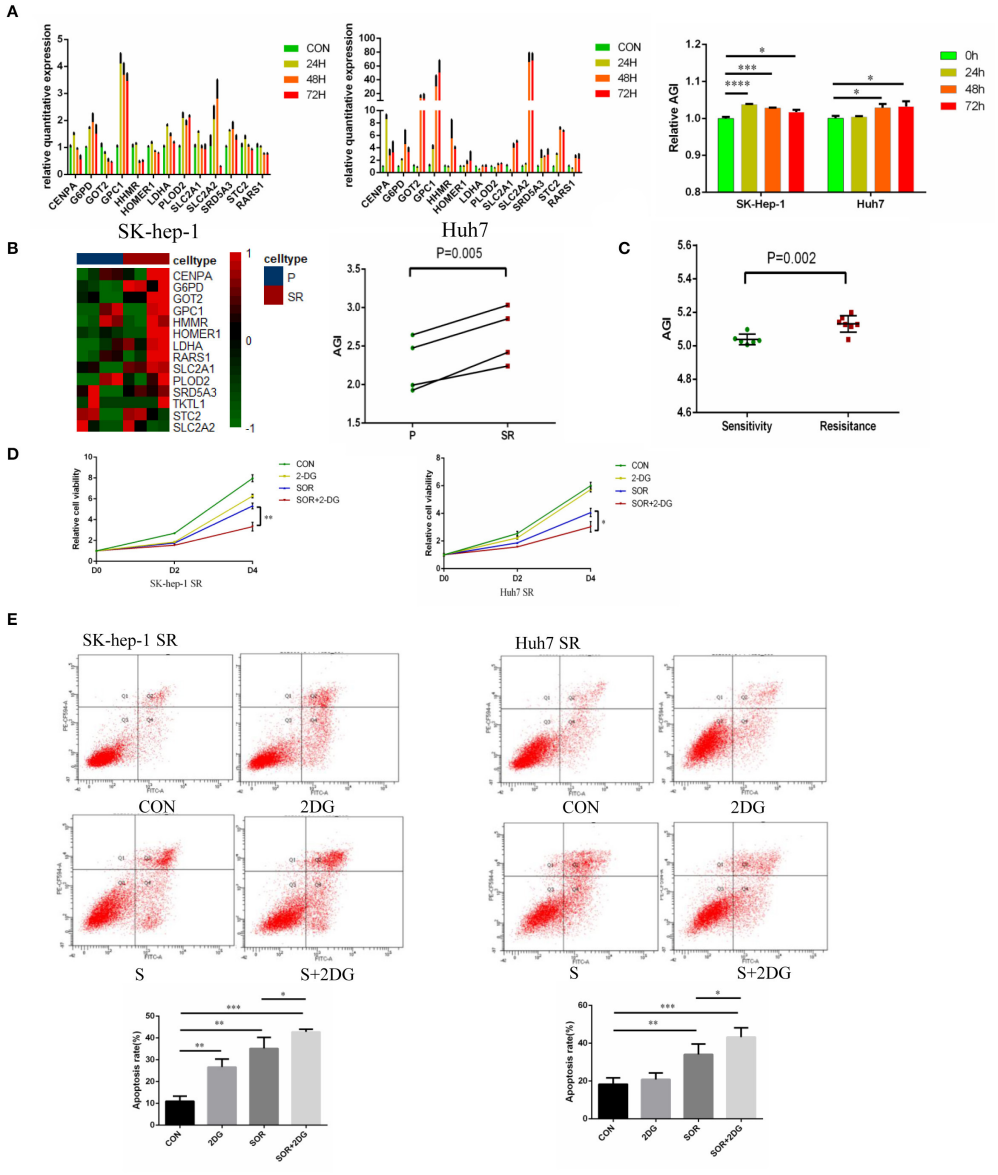
Aerobic glycolysis index (AGI) is increased in Sorafenib-resistant hepatocellular carcinoma (HCC) cells. **(A)** Relative expression of AGI-related genes in HCC cell lines (left, SK-hep-1; right, Huh7) incubated with Sorafenib (5 μM) for 24, 36, and 72 h. **(B)** Distribution of the 14 AGI-related gene expression profiles in parental and Sorafenib-resistant HCC cell lines (SK-hep-1, Huh7). **(C)** AGI of Sorafenib-sensitive and Sorafenib-resistant xenografts from the GSE73571 dataset. **(D)** Combination of 2-DG and Sorafenib resulted in significantly decreased cell viability. **(E)** Combination of 2-DG and Sorafenib enhanced the apoptosis of Sorafenib-resistant cell lines (SK-hep-1 SR and Huh7 SR). ^*^P < 0.05, ^**^P < 0.01, ^***^P < 0.001.

To further study the relationship between the AGI and Sorafenib resistance, the expression of AGI-related genes was evaluated in different cell lines (SK-hep-1 and Huh-7) at 0, 24, 48, and 72 h after Sorafenib treatment using qPCR. The levels of AGI-related genes substantially elevated following the treatment with Sorafenib, resulting in an increased AGI (Figure 7A). These results suggested that the AGI and underlying metabolic remodeling may be closely related to Sorafenib treatment. In previous studies, we reported several HCC cell lines treated with Sorafenib for long term, which could be considered as models of Sorafenib-resistant (SR) cell lines (40, 41, 46, 47). The RNA-sequencing data revealed a high AGI in SR cell lines (SK-hep-1 and Huh7) (Figure 7B). Transcript data from GSE73571 also demonstrated an elevated AGI during the acquisition of Sorafenib resistance (Figure 7C). Thus, we proposed the following hypothesis. On the one hand, tumor cells adapted to Sorafenib therapy by shifting to aerobic glycolysis. On the other hand, cells predominantly using aerobic glycolysis were also selected by Sorafenib. Both of these processes resulted in cells with enhanced aerobic glycolysis activity. Because the AGI is applied as a marker of aerobic glycolysis signaling activity, we speculate that the inhibition of aerobic glycolysis may enhance the sensitivity of Sorafenib. A combination of Sorafenib and 2-deoxy-D-glucose (2-DG), a commonly used inhibitor of aerobic glycolysis *in vitro*, was added to cell cultures, resulting in the inhibition of cell proliferation (Figure 7D) and an increased fraction of apoptotic cells (Figure 7E).

## Discussion

In the present study, we developed and validated an aerobic glycolysis-related gene signature (named the AGI) to predict the prognosis and Sorafenib sensitivity in patients with HCC. This AGI showed high accuracy in detecting HCC tumors and normal tissues. A low AGI score was significantly associated with early tumor stages, good differentiation, and better OS and DFS in a series of cohorts. Interestingly, the AGI score was correlated with the sensitivity of HCC cell lines to Sorafenib. More importantly, we demonstrated that the AGI could predict the response of patients to Sorafenib using data from clinical trials. Additionally, we observed that the AGI was elevated during the acquisition of Sorafenib resistance, which provides useful information for the development of a potential strategy to enhance Sorafenib sensitivity.

Aerobic glycolysis is a common biological process by which cancer cells tend to produce ATP by decomposing glucose or glycogen into lactic acid at a higher pace despite the presence of abundant oxygen. As an indicator of tumors, aerobic glycolysis activity is regulated by transcription factors, glucose transporters, and key enzymes of glucose metabolism. More importantly, aerobic glycolysis is related to multiple key cell signaling pathways, including PI3K/Akt, mTOR, and AMPK, and tightly associated with various cellular activities, including cell proliferation and EMT. In our study, the AGI was derived from a model consisting of genes encoding glucose transporters (SLC2A1, SLC2A2), key enzymes of glucose metabolism (G6PD, LDHA), and several other genes related to glycolysis (GPC1, HMMR, PLOD2, GOT2, STC2) (48–54), thereby supporting the use of the AGI as a marker of aerobic glycolysis activity. Several important genes related to glucose metabolism including HK2, PFK, and PKM2 are missed in the model of AGI, which had been reported to be associated with poor prognosis of HCC (55–57). The AGI was constructed using a bioinformatics method called LASSO regression. All the genes related to glycolysis were included as factors, and the LASSO regression selected factors to construct a model with minimal bias and acceptable reliability. Although HK2, PFK, and PKM2 were not in the model of AGI, we have calculated and found significant correlation between AGI and these genes. Elevated aerobic glycolysis activity was reported to result in a poor prognosis of multiple solid tumors including HCC (45, 58–60). Furthermore, several microRNAs, such as miR-383, miR-142-3p, and miR-100-5p, were reported to target LDHA, which subsequently inhibited cell proliferation, invasion, and glycolysis (61–63). Shang et al. reported that the transcription factor FOXM1 promoted glycolysis by transactivating SLC2A1 expression (64). In a respective cohort of 192 patients, the glucose transporter GLUT1 was significantly upregulated in HCC tumor tissues and was an independent risk factor of poor OS and relapse-free survival (24). Lu et al. reported that elevated G6PD expression contributed to the enhanced migration and invasion of HCC cells by inducing EMT (65), which was consistent with the correlation between the AGI and EMT signaling in the present study.

A positive correlation between aerobic glycolysis activity and Sorafenib resistance in both HCC cell lines and patients was observed in this study. Patients with a high pretreatment AGI tended to develop resistance to Sorafenib. Previously, Ma et al. and Li et al. found that increased aerobic glycolysis enhanced Sorafenib resistance in both HCC cell lines and xenografts (36, 66, 67). Key enzymes and transcription factors involved in aerobic glycolysis contributed to Sorafenib resistance, through reprogramming and redox adaptation (68), interacting with voltage-dependent anion channel (VDAC) and subsequently inhibiting mitochondrial apoptosis (69, 70) and increasing the expression of HIF-1α and c-Myc, thereby activating various cellular signals related to drug resistance (71, 72). In our previous review, we believed that Sorafenib resistance was associated with complex mechanisms, including metabolic remodeling, microenvironmental interplay, cellular signaling changes, genomic instability, and cancer stem cells (20). In our study, the levels of AGI-related genes substantially elevated following the treatment with Sorafenib, suggesting metabolic switch of glucose metabolism. Tesori et al. also reported metabolic shift toward glycolysis in HCC cells treated with Sorafenib in 48 h (31). Another study by Fiume et al. found that Sorafenib could hinder oxidative phosphorylation and stimulate aerobic glycolysis (32). Enhanced aerobic glycolysis activity was observed during the acquisition of Sorafenib resistance and reflected as an increased AGI. Thus, we speculate that the inhibition of aerobic glycolysis may enhance the sensitivity of Sorafenib. By inhibiting aerobic glycolysis activity, 2-DG resensitized HCC cells to Sorafenib therapy. Furthermore, several drugs targeting glycolysis-related factors such as Metformin, Aspirin, Genistein, Simvastatin, and Proanthocyanidin B2 have been shown to be effective in reversing Sorafenib resistance (33, 34, 36, 55, 70), indicating the aerobic glycolysis pathway as a promising target for exploring new therapies.

Notably, the present study highlighted that the AGI is a reliable biomarker in predicting the response to Sorafenib therapy. To date, several clinical and biological biomarkers have been proposed to evaluate responses to Sorafenib. The GIDEON trial revealed that patients with preserved liver function exhibited better OS after treatment with Sorafenib (73). Similarly, clinical characteristics such as Barcelona Clinic Liver Cancer (BCLC) stage, viral status, and Sorafenib-related adverse events were predictive of better survival (74, 75). As for biological biomarkers, Miyahara et al. reported that high levels of serum cytokines at baseline predicted poor outcomes in HCC patients treated with Sorafenib therapy (76). Arao et al. demonstrated that FGF3/FGF4 amplification was observed in 30% of HCC patients responding to Sorafenib (77). Recently, several exploratory studies investigated the roles of microRNAs in Sorafenib resistance and reported that several upregulated/downregulated microRNAs, long non-coding RNAs (lncRNAs), and circular RNAs (circRNAs) were predictive biomarkers of survival outcomes following Sorafenib therapy (46, 47, 78–80). Different from previous studies, the present study directly evaluated the performance of the AGI in predicting Sorafenib responses using the ROC curve and observed an optimistic AUC of 0.88, which may be more accurate and suitable in clinical practice.

The present study has several limitations. First, although the available datasets with requisite gene transcript data and clinical and treatment outcome information were all included, the predictive effectiveness of the AGI was evaluated in only a few datasets. A more careful examination is required to further confirm the accuracy of the AGI using larger and multicenter clinical cohorts in the future. Second, the AGI was derived from a model of 14 gene transcripts including several genes with very low weight or minimal detectability. A simplified AGI with fewer key genes is required to improve the robustness and clinical utility of this model. Third, the AGI was demonstrated to be essential in Sorafenib resistance and associated with various tumor hallmarks. However, several genes used to construct the AGI have not yet been reported as prognostic factors of HCC and biomarker of Sorafenib, and their underlying mechanism remains unknown.

In conclusion, we developed a gene signature based on aerobic glycolysis-related genes by integrating several transcriptomic profiles, which showed great promise for predicting prognosis and the response of HCC to Sorafenib. The AGI described in our study can be developed as a predictive biomarker for Sorafenib therapy.

## Data Availability Statement

Publicly available datasets were analyzed in this study. This data can be found here: FPKM RNA-Seq data and clinical information of TCGA LIHC was downloaded from the UCSC Cancer Browser (https://xenabrowser.net/datapages). Gene mutation data and GISTIC 2.0 segmentation scores as well as threshold copy number calls for the TCGA LIHC samples were acquired from the cBioPortal (http://www.cbioportal.org). RNA-seq and clinical data of the LIRI-JP cohort was downloaded from the HCCDB (http://lifeome.net/database/hccdb/download.html). The GSE14520, GSE25097, GSE36376, GSE64041, GSE76427, GSE109211, and GSE73571 expression profile was obtained from the GEO database (https://www.ncbi.nlm.nih.gov/geo/). The proteomics data of TCGA LIHC was downloaded from the TCPA database (https://www.tcpaportal.org/tcpa/index.html). Drug sensitivity data of HCC cell lines was obtained from the Genomics of Drug Sensitivity in Cancer (GDSC) database (https://www.cancerrxgene.org) and Cancer Cell Line Encyclopedia (CCLE) database (https://portals.broadinstitute.org/ccle/data).

## Ethics Statement

The studies involving human participants were reviewed and approved by Institutional Review Board of the Sir Run Run Shaw Hospital of Zhejiang University. The patients/participants provided their written informed consent to participate in this study.

## Author Contributions

YP and G-yH: conceptualization. YP and SJ: data curation. YP, G-yH, SJ, and S-jX: formal analysis. X-jC, J-jX, Y-hX, and L-xC: funding acquisition. Z-jL and Q-jM: investigation. YP, Q-jM, and J-jX: methodology. X-jC and J-jX: project administration. YP, G-yH, and S-jX: software. X-jC, J-jX, and Y-hX: supervision, writing review and editing. YP, JZ, L-xC, and Z-jL: validation. YP: visualization. YP and HM: writing original draft. All authors contributed to the article and approved the submitted version.

## Conflict of Interest

The authors declare that the research was conducted in the absence of any commercial or financial relationships that could be construed as a potential conflict of interest.
