# Supplementary material for: Development of an Aerobic Glycolysis Index for Predicting the Sorafenib Sensitivity and Prognosis of Hepatocellular Carcinoma
Source: Front Oncol. 2021 May 18;11:637971. doi: 10.3389/fonc.2021.637971 (PMC8169983; doi:10.3389/fonc.2021.637971)
Supplement: Supplementary file 1 [file Table_1.docx]

Table 1 The abbreviations list and their expansions for the glycolytic genes

| Symbol | Full Name |
| --- | --- |
| SLC2A1 | solute carrier family 2 member 1 |
| SLC2A2 | solute carrier family 2 member 2 |
| G6PD | glucose-6-phosphate dehydrogenase |
| LDHA | lactate dehydrogenase A |
| GPC1 | glypican 1 |
| HMMR | hyaluronan mediated motility receptor |
| PLOD2 | procollagen-lysine,2-oxoglutarate 5-dioxygenase 2 |
| GOT2 | glutamic-oxaloacetic transaminase 2 |
| STC2 | stanniocalcin 2 |
| CENPA | centromere protein A |
| RARS1 | arginyl-tRNA synthetase 1 |
| HOMER1 | homer scaffold protein 1 |
| SRD5A3 | steroid 5 alpha-reductase 3 |
| TKTL1 | transketolase like 1 |
| PFKFB3 | 6-phosphofructo-2-kinase/fructose-2,6-biphosphatase 3 |
| PFKP | phosphofructokinase, platelet |
| HK2 | hexokinase 2 |
| PKM2 | pyruvate kinase M1/2 |

Table 2 Summary of Clinical pathological parameters of included datasets

| Clinical pathological parameters | TCGA(n=371) | LRI-JP(n=203) | GSE14520(n=242) | SRRSH set(n=102) |
| --- | --- | --- | --- | --- |
| Age (mean ± SD) | 59.44±13.52 | 67±13 | 50.84±10.89 | 61.52±11.67 |
| Gender(M/F) | 250/121 | 153/50 | 211/31 | 78/24 |
| Tumor Stage |  |  |  |  |
| I | 171 | 33 | 96 | 72 |
| II | 86 | 96 | 78 | 5 |
| III | 85 | 59 | 51 | 18 |
| IV | 5 | 15 | 0 | 7 |
| NA | 24 | 0 | 17 | 0 |

SD standard deviation, M male, F female, NA not available.

Table 3 Primer sequences of glycolysis related genes

| Gene | Primer sequences |
| --- | --- |
| LDHA | F: TTGACCTACGTGGCTTGGAAG |
|  | R: GGTAACGGAATCGGGCTGAAT |
| STC2 | F: ACAGGTTCGGCTGCATAAGC |
|  | R: GAGGTCCACGTAGGGTTCG |
| GPC1 | F: TGAAGCTGGTCTACTGTGCTC |
|  | R: CCCAGAACTTGTCGGTGATGA |
| TKTL1 | F: ACAAGCAGTCAGATCCAGAGA |
|  | R: TAGCTGGCCCTGTCGAAGTA |
| SLC2A1 | F: ATTGGCTCCGGTATCGTCAAC |
|  | R: GCTCAGATAGGACATCCAGGGTA |
| SLC2A2 | F: GCCTGGTTCCTATGTATATCGGT |
|  | R: GCCACAGATCATAATTGCCCAAG |
| SRD5A3 | F: TGGCTGCACAGCTTACGAAG |
|  | R: TCAGCACAGTTAGGCCAACAA |
| PLOD2 | F: CATGGACACAGGATAATGGCTG |
|  | R: AGGGGTTGGTTGCTCAATAAAAA |
| G6PD | F: CGAGGCCGTCACCAAGAAC |
|  | R: GTAGTGGTCGATGCGGTAGA |
| HMMR | F: AACAAGCTGAAAGGCTGGTCA |
|  | R: GGGTATGAGCAGCACTACTTTT |
| GOT2 | F: AGCCTTACGTTCTGCCTAGC |
|  | R: AAACCGGCCACTCTTCAAGAC |
| RARS1 | F: ACTGTGGCTGTTTAGGAGCTT |
|  | R: ACCTCTTGTAGGCGGCTAATAA |
| CENPA | F: TTCCTCCCATCAACACAGTCG |
|  | R: CACACCACGAGTGAATTTAACAC |
| HOMER1 | F: AGAAGCTGCTCGACTAGCAAA |
|  | R: CCCGTTGATACTTTCCGGTGT |
| ACTB | F: GGCTGTATTCCCCTCCATCG |
|  | R: CCAGTTGGTAACAATGCCATGT |

Table 4 GSEA analysis of hallmark gene sets between High and low AGI group

|  | NES | p.adjust |
| --- | --- | --- |
| HALLMARK_G2M_CHECKPOINT | 2.47289 | 0.006165 |
| HALLMARK_E2F_TARGETS | 2.398493 | 0.006165 |
| HALLMARK_ALLOGRAFT_REJECTION | 2.009245 | 0.006165 |
| HALLMARK_MITOTIC_SPINDLE | 1.941174 | 0.006165 |
| HALLMARK_INFLAMMATORY_RESPONSE | 1.837388 | 0.006165 |
| HALLMARK_MYC_TARGETS_V1 | 1.799879 | 0.006165 |
| HALLMARK_EPITHELIAL_MESENCHYMAL_TRANSITION | 1.701867 | 0.006165 |
| HALLMARK_IL6_JAK_STAT3_SIGNALING | 1.665418 | 0.006165 |
| HALLMARK_TNFA_SIGNALING_VIA_NFKB | 1.586076 | 0.006165 |
| HALLMARK_SPERMATOGENESIS | 1.556085 | 0.010608 |
| HALLMARK_MTORC1_SIGNALING | 1.554346 | 0.006165 |
| HALLMARK_GLYCOLYSIS | 1.448352 | 0.013966 |
| HALLMARK_ESTROGEN_RESPONSE_LATE | 1.426576 | 0.021552 |
| HALLMARK_IL2_STAT5_SIGNALING | 1.412327 | 0.029036 |
| HALLMARK_APICAL_JUNCTION | 1.38977 | 0.037795 |
| HALLMARK_PEROXISOME | -1.91221 | 0.021368 |
| HALLMARK_OXIDATIVE_PHOSPHORYLATION | -2.07067 | 0.024272 |
| HALLMARK_COAGULATION | -2.20143 | 0.021552 |
| HALLMARK_ADIPOGENESIS | -2.20676 | 0.024272 |
| HALLMARK_FATTY_ACID_METABOLISM | -2.5452 | 0.024272 |
| HALLMARK_XENOBIOTIC_METABOLISM | -2.84849 | 0.024272 |
| HALLMARK_BILE_ACID_METABOLISM | -2.90396 | 0.021552 |

NES Normalized Enrichment Score.
