# Supplementary material for: Development of an Aerobic Glycolysis Index for Predicting the Sorafenib Sensitivity and Prognosis of Hepatocellular Carcinoma
Source: Front Oncol. 2021 May 18;11:637971. doi: 10.3389/fonc.2021.637971 (PMC8169983; doi:10.3389/fonc.2021.637971)
Supplement: Supplementary file 2 [file Data_Sheet_1.PDF]

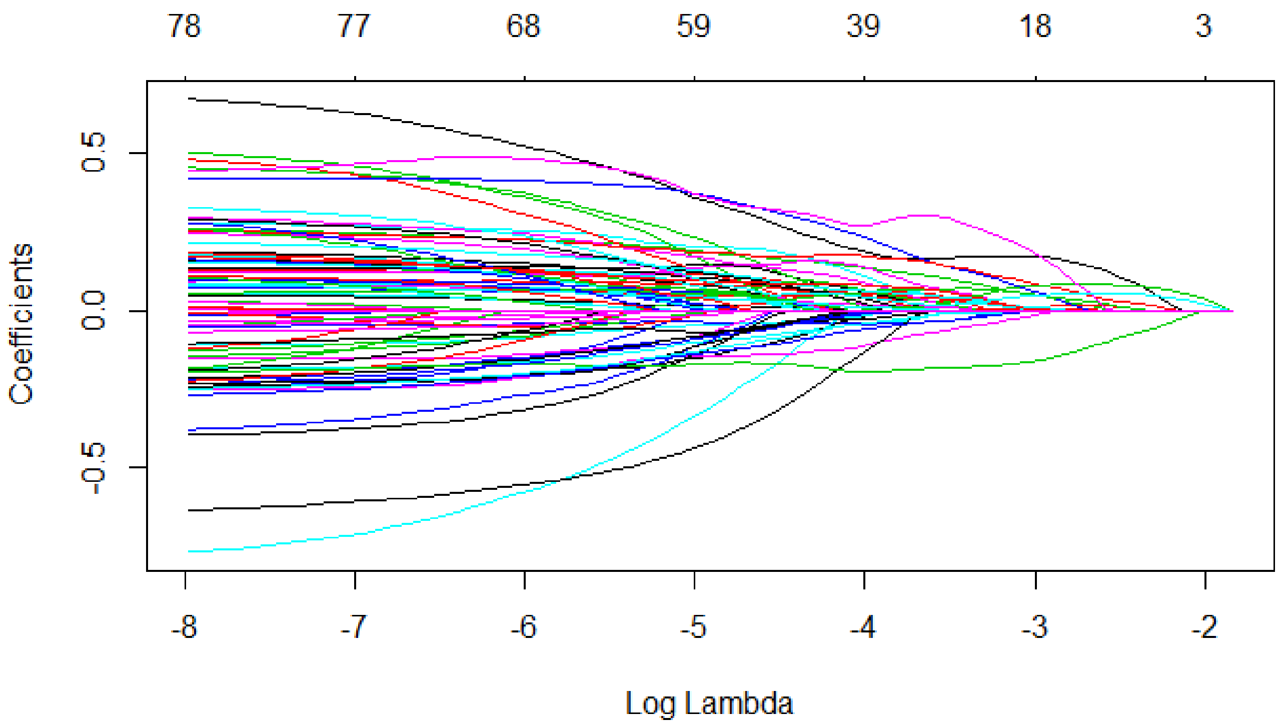

Supplementary Figure 1 LASSO coefficient profiles of the 80 genes in TCGA-LIHC

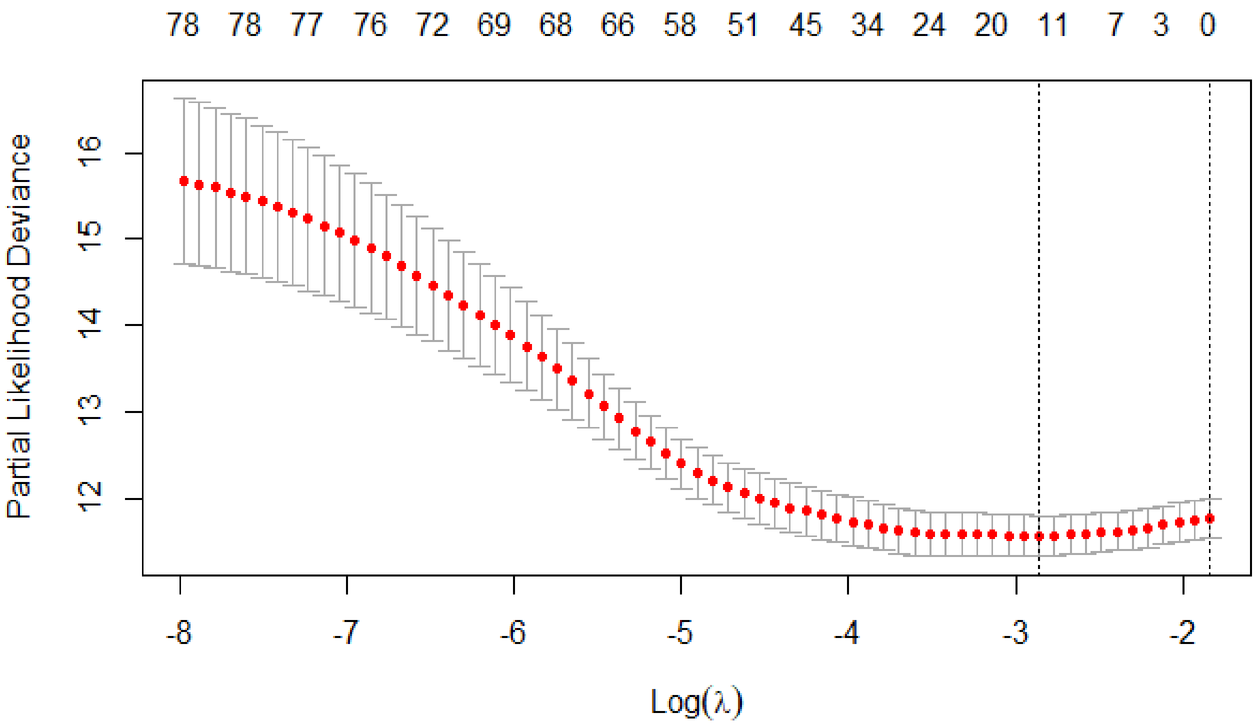

Supplementary Figure 2 The coefficient profile plot was generated against the log (lambda) sequence. Selection of the optimal parameter (lambda) in the LASSO model for TCGA dataset

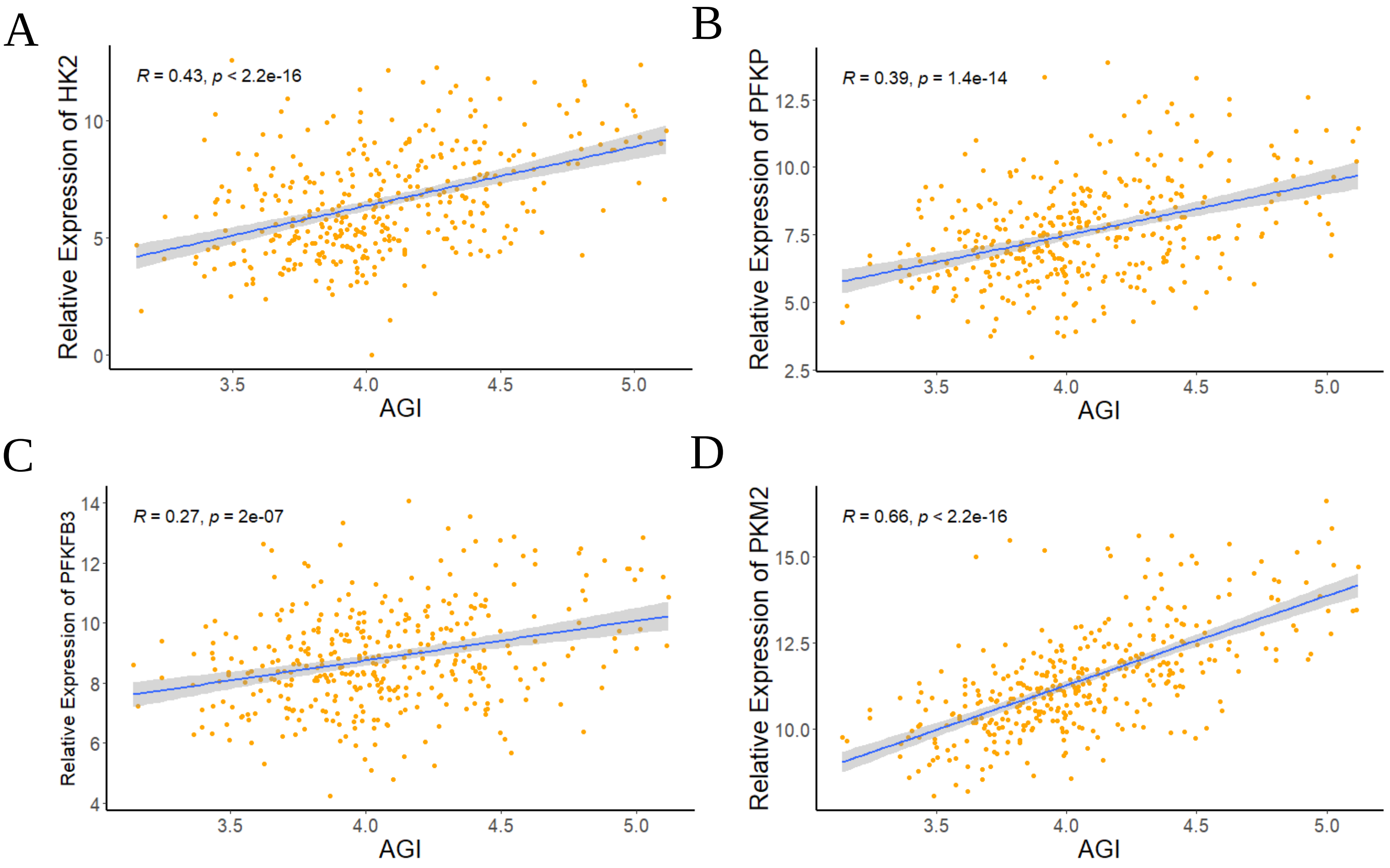

Supplementary Figure 3 Association between the AGI and genes related to glucose metabolism. **(A)** HK2. **(B)** PFKP. **(C)** PFKPB3. **(D)** PKM2.

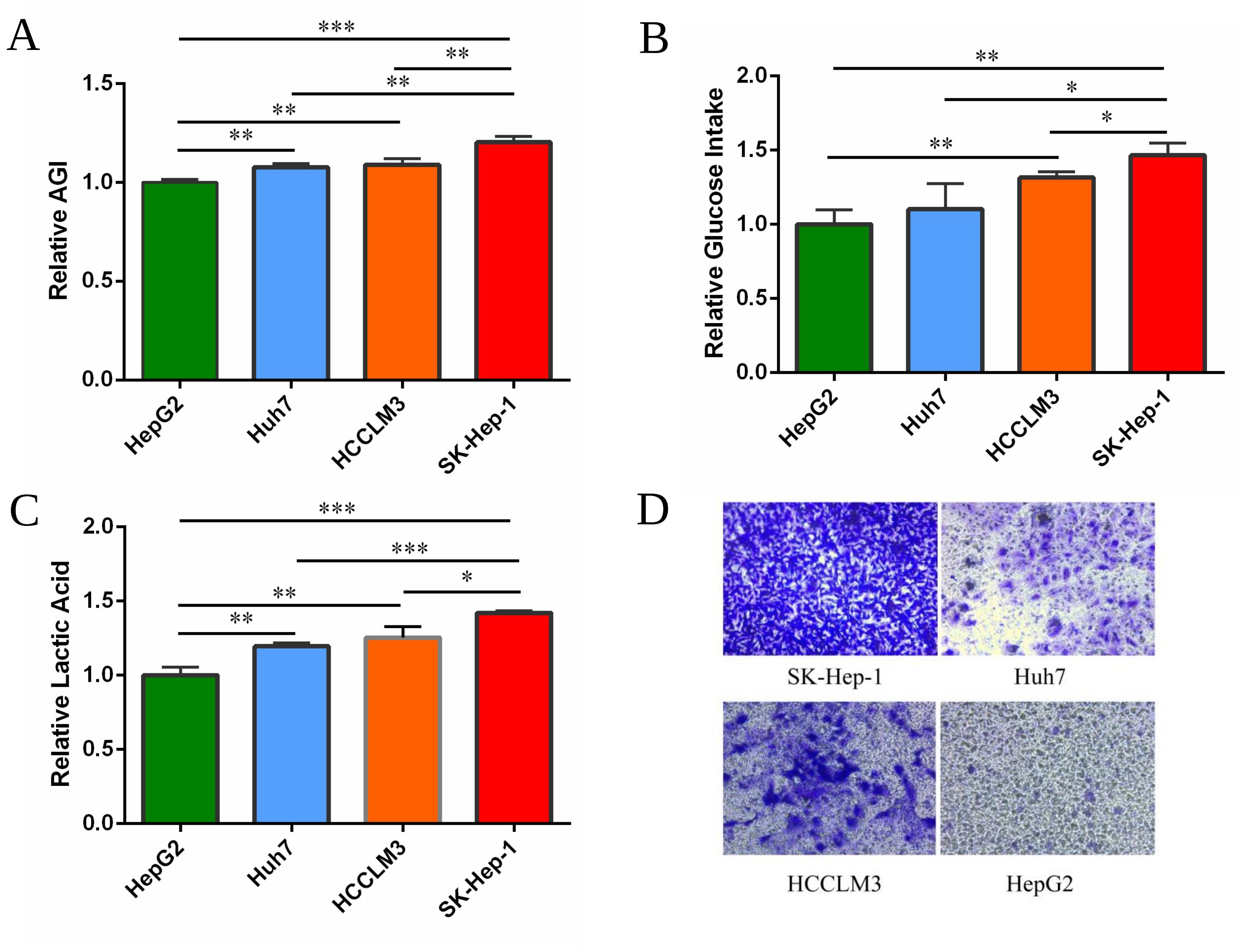

Supplementary Figure 4 AGI, glucose intake, lactic acid level and cell migration in different cell lines. **(A)** AGI. **(B)** glucose intake. **(C)** lactic acid level. **(D)** cell migration.

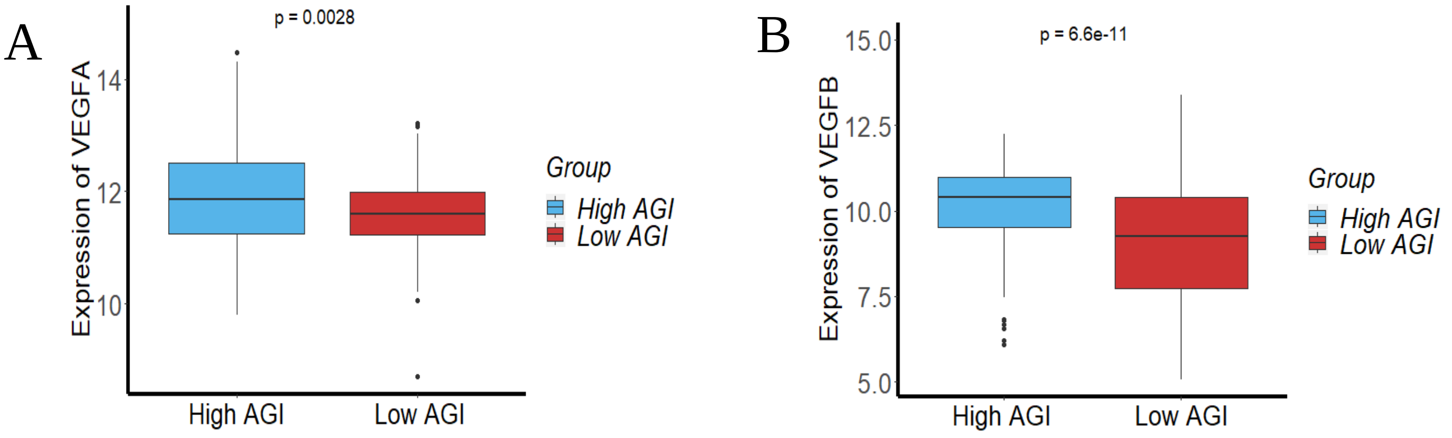

Supplementary Figure 5 comparison of genes related to angiogenesis between the high- and low- AGI groups. **(A)** VEGFA. **(B)** VEGFB.
